# 

**Published:** 1879-07

**Authors:** 


CONTENTS:

ARTICLE I.—Treatment of the Dental Pulp. (Continued) By Arthur M. Rice, D. D. S. 98
ARTICLE II.—Anæsthesia and Anæsthetics.—Criticism of an Address by Dr. Jno. J. Caldwell. By Martin P. Scott, M. D.,.... 102
ARTICLE III.—Scientific Hysteria.... 107
ARTICLE IV.—The Relations of Dentistry to Medicine. By Prof. Jas. B. Hodgkin, D. D. S.... 108
ARTICLE V.—Corundum.... 116
ARTICLE VI.—Applications of Celluloid.... 123
ARTICLE VII.—Recent Experiments with Laughing Gas.... 127
ARTICLE VIII.—The Indiana Dental Law.... 129
ARTICLE IX.—Dental Associations—Virginia State Dental Association.... 131
Nationa. Dental Association.... 132
South Carolina State Dental Association.... 133
American Dental Association.... 134
Georgia State Dental Society.... 134
American Dental Convention.... 135

EDITORIAL, ETC.

Indiana Medical Protective Law.... 136

Loss & Gain.... 137

Storms & Neuralgia.... 139

Orkney Springs, Virginia.... 140

MONTHLY SUMMARY.

The Woman Doctor Question.... 140
How to Avoid Leaving Scars.... 140
Personal Similarities & Dissimilarities.... 141
Poisoning by Chlorate of Potash.... 141
Prevention of Fatal Accidents from Anæsthetics.... 142
A New Insect Powder.... 142
A Very Large Salivary Calculus.... 143
Replanting Teeth.... 143
Registration of Dentists in England.... 143
Chromic Pharynzitis.... 143
Ergotine for Neuralgia.... 144
Treatment of Ulcers.... 144
Whooping Cough and Ulceration of the Frænum.... 144
Chloroform Narcosis.... 144
A Perfumed Solution of Iodoform.... 144
Freckles.... 144

